# Supplementary material for: The Construction and Exploration of a Comprehensive MicroRNA Centered Regulatory Network in Foxtail Millet (Setaria italica L.)
Source: Front Plant Sci. 2022 May 6;13:848474. doi: 10.3389/fpls.2022.848474 (PMC9121102; doi:10.3389/fpls.2022.848474)
Supplement: Supplementary file 2 [file Data_Sheet_2.zip › Sit-miR5179_Seita.4G277600.1_620_TPlot.pdf]

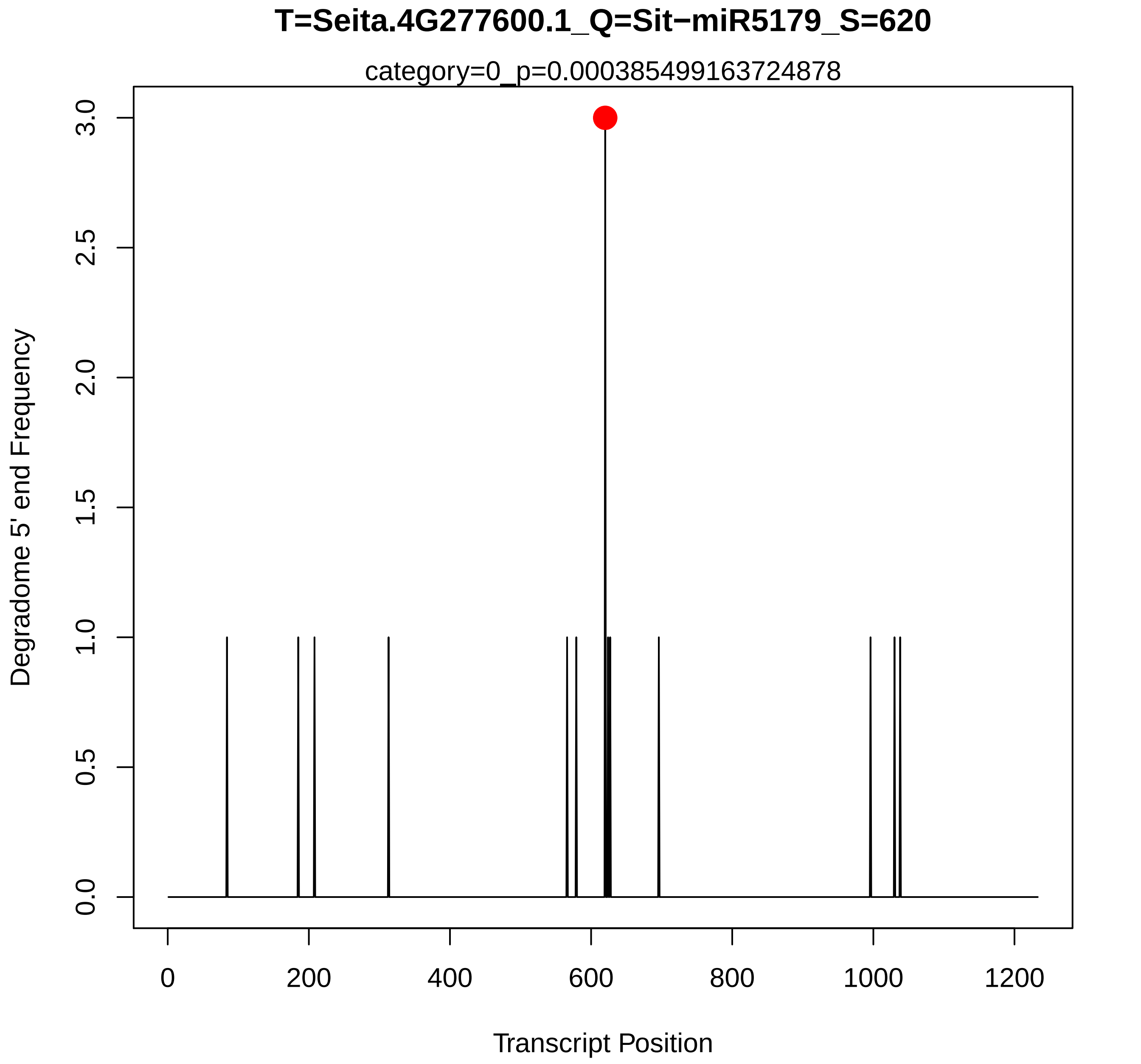

T=Seita.4G277600.1_Q=Sit−miR5179_S=620
category=0_p=0.000385499163724878
Degradome 5' end Frequency
0.0
0.5
1.0
1.5
2.0
2.5
3.0
0
200
400
600
800
1000
1200
Transcript Position
